# Association between Body Water Status and Acute Mountain Sickness

**DOI:** 10.1371/journal.pone.0073185

**Published:** 2013-08-27

**Authors:** Hannes Gatterer, Maria Wille, Martin Faulhaber, Henry Lukaski, Andreas Melmer, Christoph Ebenbichler, Martin Burtscher

**Affiliations:** 1 Department of Sport Science, Medical Section, University Innsbruck, Innsbruck, Austria; 2 Department of Physical Education, Exercise Science & Wellness, University of North Dakota, Grand Forks, North Dakota, United States of America; 3 Department of Internal Medicine I, Innsbruck Medical University, Innsbruck, Austria; University of Las Palmas de Gran Canaria, Spain

## Abstract

**Purpose:**

The present study determined the association between body fluid variation and the development of acute mountain sickness (AMS) in adults.

**Methods:**

Forty-three healthy participants (26 males and 17 females, age: 26±6 yr, height: 174±9 cm, weight: 68±12 kg) were passively exposed at a FiO_2_ of 12.6% (simulated altitude hypoxia of 4500 m, PiO_2_ = 83.9 mmHg) for 12-h. AMS severity was assessed using the Lake Louise Score (LLS). Food and drink intakes were consumed *ad libitum* and measured; all urine was collected. Before and after the 12-h exposure, body weight and plasma osmolality were measured and whole-body bioimpedance analysis was performed.

**Results:**

The overall AMS incidence was 43% (38% males, 50% females). Participants who developed AMS showed lower fluid losses (3.0±0.9 vs. 4.5±2.0 ml/kg/h, p = 0.002), a higher fluid retention (1.9±1.5 vs. 0.6±0.8 ml/kg/h, p = 0.022), greater plasma osmolality decreases (−7±7 vs. −2±5 mOsm/kg, p = 0.028) and a larger plasma volume expansion (11±10 vs. 1±15%, p = 0.041) compared to participants not developing AMS. Net water balance (fluid intake – fluid loss) and the amount of fluid loss were strong predictors whether getting sick or not (Nagelkerkes r^2^ = 0.532). The LLS score was related to net water balance (r = 0.358, p = 0.018), changes in plasma osmolality (r = −0.325, p = 0.033) and sodium concentration (r = −0.305, p = 0.047). Changes in the impedance vector length were related to weight changes (r = −0.550, p<0.001), fluid intake (r = −0.533, p<0.001) and net water balance (r = −0.590, p<0.001).

**Conclusions:**

Participants developing AMS within 12 hours showed a positive net water balance due to low fluid loss. Thus measures to avoid excess fluid retention are likely to reduce AMS symptoms.

## Introduction

Rapid ascents of non-acclimatized mountaineers to altitudes above 2,500 m are associated with the development of acute mountain sickness (AMS).This normally self-limiting syndrome is characterized by non-specific symptoms such as headache, dizziness, nausea, vomiting, loss of appetite, fatigue, and insomnia [1], [2]. Symptoms of AMS typically appear 6–12 h after arrival at high altitude [3]. Risk factors associated with the development of AMS include the absolute altitude reached [4], [5], [6], the rate of ascent [4], [7] and the individual susceptibility [1], [3], [7], [8]. Controversy exists whether other factors such as low fluid intake and dehydration [6], [9], [10] or overhydration [11], [12] promote AMS because of the lack of agreement in other studies [13], [14], [15]. These divergent findings may be explained by varied experimental designs (e.g., laboratory vs. field studies, resting vs. exercise conditions) and the lack of well controlled studies [15]. Less controversy exists on the impact of a decreased diuresis and fluid retention on AMS development [14],[16],[17],[18]. The majority of studies point towards augmentation of AMS incidence by such disturbances even though prospective investigations have not always revealed a consistent difference in fluid balance between subjects developing AMS and those remaining well [19]. Controversy may arise due to the methodological divergences mentioned above. Additionally, changes in fluid balance have been reported to be different between hypobaric hypoxia vs. normobaric hypoxia conditions suggesting that both, reduced barometric pressure and oxygen partial pressure contribute to fluid retention [20]. However, the effect of different conditions on the AMS development has not been established yet. Thus, assessment of fluid homeostasis during passive normobaric hypoxia exposure (i.e., controlled normobaric hypoxic conditions, without the influence of exercise, hypobaria, cold) might help to clarify the pathophysiological relevance of hypoxia on the hydration status and the concomitant AMS development.

A noninvasive method to monitor body fluid variation is the bioelectrical impedance analysis (BIA). BIA provides quantitative estimates of total, as well as intra- and extracellular water content based on regression equations. The R-Xc graph method on the other hand uses solely resistance (R) and reactance (Xc) normalized for standing height (bioimpedance vector analysis or BIVA) [21] and enables the classification of the hydration status independently of regression predictions which is considered the major weakness described for conventional BIA [22]. Piccoli et al. suggested it to be a useful tool in the planning of climber's appropriate hydration and fluid intake during ascent to altitude [23].

As mentioned before, only one study analysed the effects of passive normobaric hypoxia exposure on fluid balance. The authors suggested extracellular fluid shifts to be involved in AMS development, but did not establish the exact nature of its impact [20]. Therefore, the present study aimed to assess the relationship between body fluid shifts/variation and the development of AMS during the early hours of a standardized passive normobaric hypoxia exposure in a large sample of men and women. According to Burtscher et al. there is no clear evidence for sex differences in the response to hypoxia [24] but as AMS incidence was sometimes reported to differ between sexes [25] perhaps because of the effects of sex hormones on ventilatory control [26] a comparison between men and women was made. We hypothesized that net fluid retention and extracellular fluid shifts during acute exposure to simulated hypoxia are associated with development of AMS with no differences between sexes and that we would be able to demonstrate this by the use of BIVA in combination with measures of conventional hydration markers (e.g., plasma osmolality).

## Materials and Methods

### Participants

Participants were recruited by advertisements on the homepage of the Austrian Alpine Association and by information via the mailing list of the University of Innsbruck. Exclusion criteria were reported cardiovascular, respiratory, neurological and psychiatric diseases, migraine, chronic headache, smoking, permanent residence at altitudes exceeding 1,000 m, an overnight stay at altitudes >2,500 m in the previous month, or exposure above >2,500 m two wk prior to the 12- hr hypoxic exposure. Fifty-six adults (32 males and 24 females) aged 18 to 45 yr, who fulfilled the inclusion criteria, volunteered to participate in the study. In the course of the 12-hr session, 13 participants suffered an emesis and the volume could not have been determined. Therefore, these participants were excluded from analysis, except for the calculation of the overall incidence rate. The final data set for all analysis included 43 participants, 26 males and 17 females.

Participants were instructed to abstain from all anti-inflammatory medication and nutritional supplements for two weeks prior to the exposure and from alcohol starting the day before the experiment. Caffeine was not allowed on the day of the exposure. All participants gave their written informed consent prior to the participation in the study. The study was carried out in conformity with the ethical standards laid down in the 2008 declaration of Helsinki and was approved by the ethics committee of the Medical University of Innsbruck.

### Procedures

Participants were passively exposed at a F_i_O_2_ of 12.6% (corresponding to a simulated altitude hypoxia of 4,500 m, P_i_O_2_ = 83.9 mmHg) for 12 hr. Room temperature and humidity were kept constant at 22–24°C and 23–27%, respectively. Prior to entering the hypoxic chamber participants were examined, including a medical routine check. Women were tested for pregnancy before the 12-hr hypoxic exposure. During the simulated hypoxia exposure, the same food (i.e., brown bread, cheese, boiled ham, cucumber, banana, apple, cookies and chocolate) and drinks (water and apple juice) were provided and consumed *ad libitum*. Most of the time participants seated but some activities (e.g., standing, walking and stretching) were also performed. Recumbent position or sleeping was not allowed. Measurements, described in detail in the following sections, were performed before, during or/and after the session.

### Measurements and instruments

#### Lake Louise Score (LLS) and AMS

The LLS was used to assess the incidence and severity of AMS [27]. It is a self-assessment questionnaire including five symptom complexes (headache, gastrointestinal symptoms like anorexia, nausea or vomiting, fatigue and/or weakness, dizziness and/or light headedness, difficulty sleeping); scores range from 0 to 3. The subjects self-rated their status; 0 for no discomfort, 1 for mild, 2 for moderate and 3 for severe symptoms. Because participants did not stay overnight in the hypoxic chamber, the symptom complex difficulty sleeping was not taken into account. AMS was diagnosed when the symptom headache was present and at least one other symptom, with a total score of either ≥ 3 to recognize mild diseases or AMS at an early stage or ≥ 4 to recognize a more severe diseases state [28]. The LLS at the point when leaving the chamber was taken to distinguish between AMS+ and AMS−.

#### Diuresis, insensible water loss, fluid and food intake

Urine volume, fluid and food intake (amount and composition) was recorded during the 12-hr exposure. Measuring cups were used to determine urine and fluid intake volume. For every participant the same limited number of food products was provided and food was weighted with a food scale before ingestion. Fluid content of the food was calculated by using the DGE-PC Professional software (Version 3.2, Bonn, Germany). The solid food content was calculated as the amount of food (g) minus the fluid content (ml) and was used to correct the body weight after the exposure (body weight minus solid food content). Insensible water loss (respiratory and surface water loss) was estimated to be 0.5 mL/kg/h [29]. Net water balance was calculated as fluid intake (water intake + water content of the food) minus fluid loss (urinary volume + insensible water). All volumes were normalised for body weight and exposure hours (ml/kg/h).

#### BIA, BIVA and body weight

Directly before entering and after leaving the chamber body weight was determined after urinating, wearing the same clothes and without shoes to the nearest 0.1 kg using a calibrated digital scale (Axxence easy coach, Bosch, Stuttgart, Germany). Bioimpedance analysis (BIA) was performed before and immediately after leaving the chamber using an impedance plethysmograph that emits an 800- µA, 50-kHz alternating current (BIA-101, RJL/Akern Systems, Clinton Township, MI, USA). Before performing the measurements participants were asked to empty their bladder. Measurements were taken with subjects being in a supine position for 5 minutes, according to the manufacturers guidelines. Surface, contact electrodes (Biatrodes, Akern, Florence, Italy) were placed on the right hand and foot. The position of the electrodes was marked with a permanent marker in case the electrodes went off; otherwise they were left throughout the hypoxic exposure. A comparison between newly placed electrodes and electrodes left for 12 hr was performed in 6 participants. R and Xc values did not differ between condition [R = 473±39Ω vs. 473±40Ω (ICC r = 0.996, p<0.001); Xc = 59.4±9.3Ω vs. 59.3±7.9Ω (ICC r = 0.967, p<0.001)].

Beside estimation of total body water (TBW, formula of Sun et al. [30]), extracellular water (ECW, equation developed from the manufacturer and not available) and intracellular water (ICW = TBW-ECW), BIA analyses were done according to the R-Xc graph method [21]. Measurements of R and Xc were standardized by the height of subjects (i.e., R/H and Xc/H) and were expressed in ohm/m. The combination of R (i.e., opposition to the flow of an alternating current through intra- and extra-cellular ionic solution) and Xc (i.e., capacitive component of tissue interfaces, cell membranes and organelles) yields an impedance vector (geometric relationship = arc tangent of Xc/R) [22]. The length of the vector is inversely related to TBW [22] and was calculated as the hypotenuses of individual impedance values [31]. This approach enables the classification of tissue hydration status and body cell mass (BCM) by solely considering impedance components independently of regression predictions of fluid volumes or assumptions of constant chemical composition of the fat-free body [22].

#### Haematological parameters

Venous blood samples [4 mL Z Serum Sep Clot Activator (Vacuette, Greiner Bio-One, Germany)] were taken before and at the end of the 12-hr hypoxic exposure. Blood samples were immediately centrifuged at 4000 g at 4°C for 15 min. Plasma osmolality (Posm) was measured in replicate by freezing point depression method (Fiske Micro-Osmometer, model 210; Fiske, Norwood, MA). When the outcome of the analysis differed less than 2 mOsm/kg, measurements were performed twice, otherwise a third measurement was performed. The mean of the 2 or 3 measurements was used for analysis.

Arterialized capillary blood samples (125 µL) were taken from the participant's ear lobe before, after 6 hr and immediately before leaving the chamber and were analysed immediately for sodium concentration (Na^+^), hematocrit (Hct), bicarbonate concentration (HCO_3_^−^), pH and partial pressure of oxygen and carbon dioxide (PO_2_ and PCO_2_, respectively) using the ABL80 FLEX Co-Ox, Radiometer (Denmark). The ear lobe was made hyperaemic 10 min prior to the sampling with Finalgon® (BI Pharma Thomae, Germany). Plasma volume was calculated assuming a blood volume of 77 ml/kg and changes in plasma volume (ΔPV) were calculated according to the formula of Van Beaumont [32]. Arterial oxygen saturation (SpO_2_) was measured by pulsoxymetry (Onyx II 9550, NONIN, Plymouth, MI, USA) before the capillary blood samples were taken.

### Statistical analysis

This study tested the hypothesis that altered fluid balance affects the development of AMS during simulated exposure to hypoxia. Data analyses were performed with the use of the SPSS statistical-software package (PASW Statistic 18). The chi-square test was used to calculate difference in incidence of AMS between men and women. Analysis of variance (ANOVA) for repeated measurements with 2 between-subjects factors (AMS + vs. AMS-, men vs. women) and one within-subject factor (measurement time before and after the 12-hr exposure) was performed. Unpaired student-t tests were used to determine differences between sexes and between AMS+ and AMS- for parameters of fluid balance (urinary volume, fluid intake, net water balance). Paired one sample Hotellinǵs T^2^ test were used to calculate significant vector displacements and two sampe Hotellinǵs T^2^ test were used to determine vector differences between AMS- and AMS+. Pearson correlation analysis was used to assess the relationship between measured or calculated values and difference variables (Δ) (post minus pre). A stepwise logistic regression analysis, adjusted for sex and age, with backward variable selection was applied to identify predictor hydration variables for AMS+ and AMS-. Variables included in the analysis were *Δ*Na^+^, *Δ*Posm, *Δ*vector length, *Δ* weight, fluid intake, fluid loss and net water balance. The mean of values measured after 6 hr and immediately before leaving the chamber (i.e. SpO_2_, Na^+^, HCO_3_^−^, PCO_2_, PO_2_ and pH) was used for statistical analysis. A p-value of less than 0.05 (two-tailed) was considered to indicate statistical significance. Results are expressed as mean±standard deviation (SD), 95% confidence interval (CI) and odds ratio (OR).

## Results

### AMS incidence

The AMS incidence when using a LLS cut off of ≥3 was 46.5% with no differences between sexes (50% and 41% for males and females, respectively; p = 0.571). When using a LLS cut off of ≥ 4 the AMS incidence was 26% and was similar between sexes (23% and 29% for males and females, respectively; p = 0.642). The mean LLS was 5.1±2.5 and 1.0±0.8 for AMS+ and AMS- when using the LLS cut off of ≥3 and 6.7±2.2 and 1.6±1.1 when using the cut off of ≥ 4. When participants with emesis were included the overall incidence increased to 59% or 43% for cut off ≥3 or 4, respectively (males 59% or 38% and females 58% or 50%; p = 0.938 or 0.350). Due to very severe symptoms 3 males and 3 females out of 43 participants left the chamber before the end of the 12 hr (mean stay of these participants: males = 8.6±2.4 hr, females = 7.2±2.0 hr). No sex differences were found for the fluid intake, the fluid loss and for the net water balance (p>0.05). No sex effects (interaction: time x sex) for changes in weight, Na^+^, Hct, R, Xc and vector length (p>0.05) were found, except for Posm (p<0.05). No interaction (time x sex x AMS+/−) was found for any of the analyzed variables. Because only small sex effects (see section above) were detected combined results of male and females were depicted in the following sections.

### Body hydration, AMS and explanatory measures

**Figure 1:**
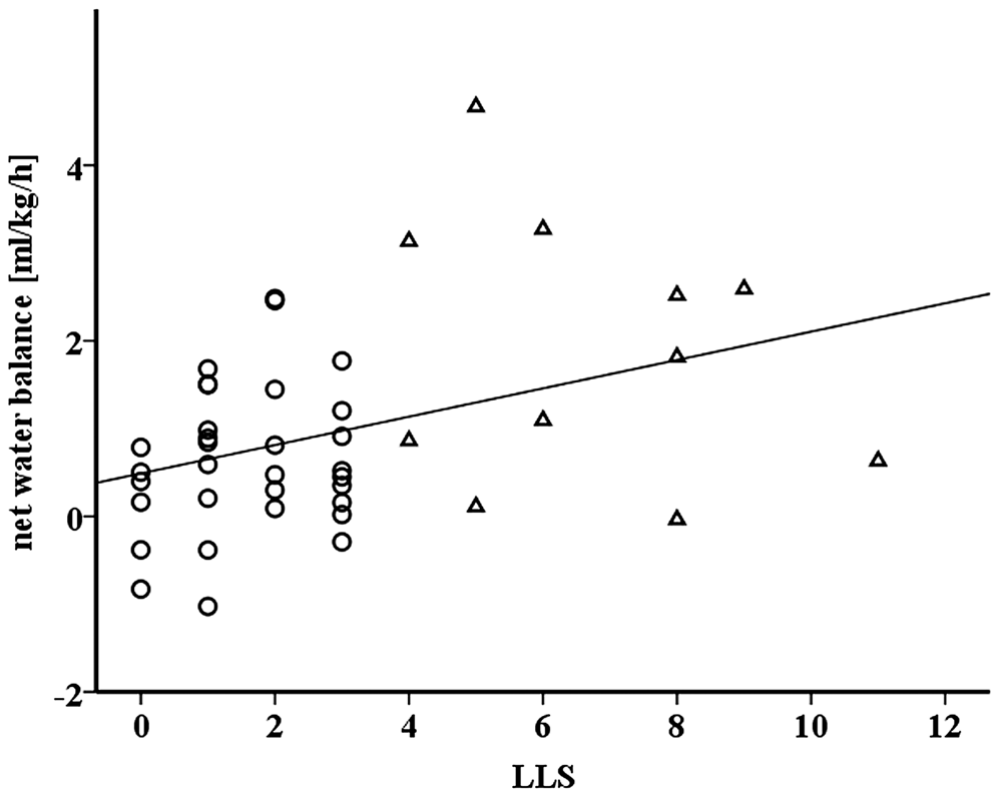
Relationship between net water balance and the Lake Louise Score (LLS) (r = 0.358, p = 0.018).

When using the LLS cut off of ≥ 3 only a trend towards significant differences between AMS− and AMS+ were found with respect to net water balance (0.7±0.9 ml/kg/h vs. 1.3±1.3 ml/kg/h, p = 0.092, respectively). No differences were found for fluid intake and fluid loss (p>0.05). Therefore in the following only the results for the more severe disease state (LLS ≥ 4) were shown. Baseline characteristics separated for sexes are shown in Table 1. AMS− and AMS+ differed in regard to fluid loss (4.5±2.0 ml/kg/h vs. 3.0±0.9 ml/kg/h for AMS− and AMS+, respectively; p = 0.002) and net water balance (0.6±0.8 ml/kg/h vs. 1.9±1.5 ml/kg/h for AMS− and AMS+, respectively; p = 0.022). No difference was found in regard to fluid intake (5.1±2.3 ml/kg/h vs. 4.9±1.4 ml/kg/h for AMS− and AMS+, respectively; p = 0.990). The regression analysis identified net water balance (1.09–8.68 CI, 3.1 OR) and fluid loss (0.29–1.05 CI, 0.55 OR) as predictor variables for the development of AMS (Nagelkerkes r^2^ = 0.532 of the final model). Sodium intake did not differ between AMS− and AMS+ (1.7±0.8 g vs. 1.7±0.8 g, respectively; p = 0.861). Table 2 shows the outcomes for the hydration parameters. There were significant interactions (time x AMS+/−) for weight and Posm (p = 0.001 and p = 0.028). ΔPV, obtained from the formula of Van Beaumount (1972) [32], showed significant differences between AMS+ and AMS− (11±10 vs. 1±15%, respectively, p = 0.041). Outcome of the correlation analysis is shown in Table 3. A significant correlation between the LLS score and the net water balance (Figure 1), Δ Posm and Δ Na^+^ was found. Table 4 shows the outcome of the ANOVA for HCO_3_^−^, SpO_2_, pH and the blood gases. There were significant interactions (time x AMS+/−) for HCO_3_^−^ (p = 0.038) and SpO_2_ values (p = 0.008). SpO_2_, HCO_3_^−^ and PCO_2_ values during the exposure were correlated to the AMS score (Table 3). HCO_3_^−^, pH, PCO_2_, and PO_2_ values during the exposure were correlated with the SpO_2_ (r = −0.473, p = 0.001; r = 0.675, p = <0.001; −0.704, p = <0.001 and r = 0.615, p = <0.001, respectively). No relationship was found for HCO_3_^−^, SpO_2_, PO_2_, pH and PCO_2_ with any parameter of body hydration.

**Table 1:** Baseline characteristics (mean±SD) for participants who developed AMS (AMS+) and participants who did not (AMS−) separated for sexes.

	all	male	female	all AMS+	all AMS−	male AMS+	male AMS−	female AMS+	female AMS−
n	43	26	17	11	32	6	20	5	12
age [years]	26.1±5.7	26.1±5.6	26.1±6.1	30±7	25±5#	30.7±7.1	24.7±4.4^t^	29.0±7.6	24.8±5.2
body weight [kg]	67.9±11.7	73.4±9.9	59.4±9.1*	70.0±13.2	67.2±11.3	76.3±13.9	72.5±8.6	62.3±7.8	58.2±9.7
body height [m]	1.74±0.09	1.79±0.07	1.66±0.06*	1.73±0.09	1.75±0.09	1.80±0.06	1.80±0.07	1.64±0.06	1.67±0.06
Posm [mOsm/kg]	297±3	298±3	296±3	297±3	297±3	296±4	298±3	297±3	295±4
Na [mmol/L]	140±2	140±2	140±2	140±2	140±2	140±2	140±2	140±1	141±2
R/height [Ω/m]	315±49	284±25	364±35*	312±44	317±51	280±28	285±24	350±19	369±40
Xc/height [Ω/m]	37±4	36±4	39±4*	37±5	37±4	35±4	36±4	40±3	39±4
vector length	318±49	286±25	366±38*	314±44	319±51	282±29	287±24	353±18	371±40

Plasma osmolarity (Posm), sodium (Na), resistance divided by body height (R/height), reactance divided by body height (Xc/height).

* indicates differences between sexes (p<0.05).

# indicates differences between AMS+ and AMS− (p<0.05)

t tendency to be different between AMS+ and AMS− (0.05<p<0.1).

**Table 2:** Changes in hydration parameters (mean±SD) over the course of the 12 hour exposure (n = 43).

	AMS+			AMS−				
	pre	post	Δ%	pre	post	Δ%	ANOVA main effect: time	ANOVA interaction: time x AMS+/−
body weight [kg]	69.9±13.2	70.6±13.4	**+1.0**	67.2±11.3	67.0±11.3	**−0.3**	**0.037**	**0.001**
Posm [mOsm/kg]	297±3	290±9	**−2.4**	297±3	295±5	**−0.7**	**<0.001**	**0.028**
Na [mmol/L]	140±2	137±4	**−2.1**	140±2	140±2	**0**	**0.001**	0.050
Hct [%]	49.8±3.6	47.3±4.0	**−5.0**	49.2±4.0	49.3±3.9	**+0.2**	0.050	0.054
PV* [L]	2.7±0.5	3.0±0.5	**+11.1**	2.6±0.4	2.6±0.5	**0**	**0.028**	0.052
TBW [L]	39.7±7.6	40.9±8.2	**+3.0**	39.4±6.7	40.3±7.0	**+2.3**	**<0.001**	0.286
ECW [L]	16.6±3.0	17.5±3.2	**+5.4**	16.6±2.1	17.2±2.4	**+3.6**	**<0.001**	0.151
ICW [L]	23.1±4.7	23.4±5.1	**+1.3**	22.8±4.9	23.1±4.8	**+1.3**	**0.005**	0.586
R/height [Ω/m]	312±44	300±48	**−3.8**	317±51	305±51	**−3.8**	**<0.001**	0.872
Xc/height [Ω/m]	37±5	35±5	**−5.4**	37±4	35±4	**−5.4**	**<0.001**	0.240
vector length	314±44	302±49	**−3.8**	319±51	307±51	**−3.8**	**<0.001**	0.852
		Δ values			Δ values			Unpaired t-test
PV [%]#		11±10			1±15			**0.041**
net water balance [ml/kg/h]		1.9±1.5			0.6±0.8			**0.022**
fluid loss [ml/kg/h]		3.0±0.9			4.5±2.0			**0.002**
fluid intake [ml/kg/h]		4.9±1.4			5.1±2.3			0.999

Plasma osmolarity (Posm), sodium (Na), hematocrit (Hct), total body water (TBW), resistance divided by body height (R/height), reactance divided by body height (Xc/height), plasma volume (PV).

* calculated from Hct assuming 77 ml/kg of blood volume.

# calculated from the formula of Van Beaumont W (1972) [32].

**Table 3:** Correlation analysis for the LLS and the explanatory variables (n = 43).

	net water balance	Δ Posm	Δ Na^+^	Δ weight	Δ Hct	SpO_2_	HCO_3_^−^	PCO_2_
LLS	0.358*	−0.325*	−0.305*	0.296^t^	−0.262^t^	−0.376*	0.388*	0.386*

Lake Louse Score (LLS), plasma osmolarity (Posm), sodium (Na), hematocrit (Hct), arterial oxygen saturation (SpO_2_), bicarbonate concentration (HCO_3_^−^), partial pressure of carbon dioxide (PCO_2_).

* p<0.05; ^t^ 0.05<p<0.1.

**Table 4:** Changes in HCO_3_^−^, SpO_2_, pH and the blood gases (mean±SD) over the course of the 12 hour exposure.

	AMS+			AMS−				
	pre	post	Δ%	pre	post	Δ%	ANOVA main effect: time	ANOVA interaction: time x AMS+/−
SpO_2_ [%]	98±2	81±5	**−17**	98±1	85±4	**−13**	**<0.001**	**0.008**
HCO_3_^−^ [mmol/L]	24.3±1.9	23.8±1.9	**−2.1**	23.8±1.6	22.4±1.4	**−5.9**	**<0.001**	**0.038**
pH	7.40±0.02	7.46±0.02	**+0.8**	7.41±0.03	7.47±0.03	**+0.8**	**<0.001**	0.673
PO_2_ [mmHg]	73.1±5.6	35.9±3.8	**−50.9**	77.0±6.9	38.2±3.8	**−50.4**	**<0.001**	0.413
PCO_2_ [mmHg]	39.6±2.7	34.2±3.2	**−13.6**	37.9±3.8	31.5±3.2	**−16.9**	**<0.001**	0.393

Arterial oxygen saturation (SpO_2_), bicarbonate concentration (HCO_3_^−^), partial pressure of oxygen and carbon dioxide (PO_2_ and PCO_2_).

### BIVA and body hydration

**Figure 2:**
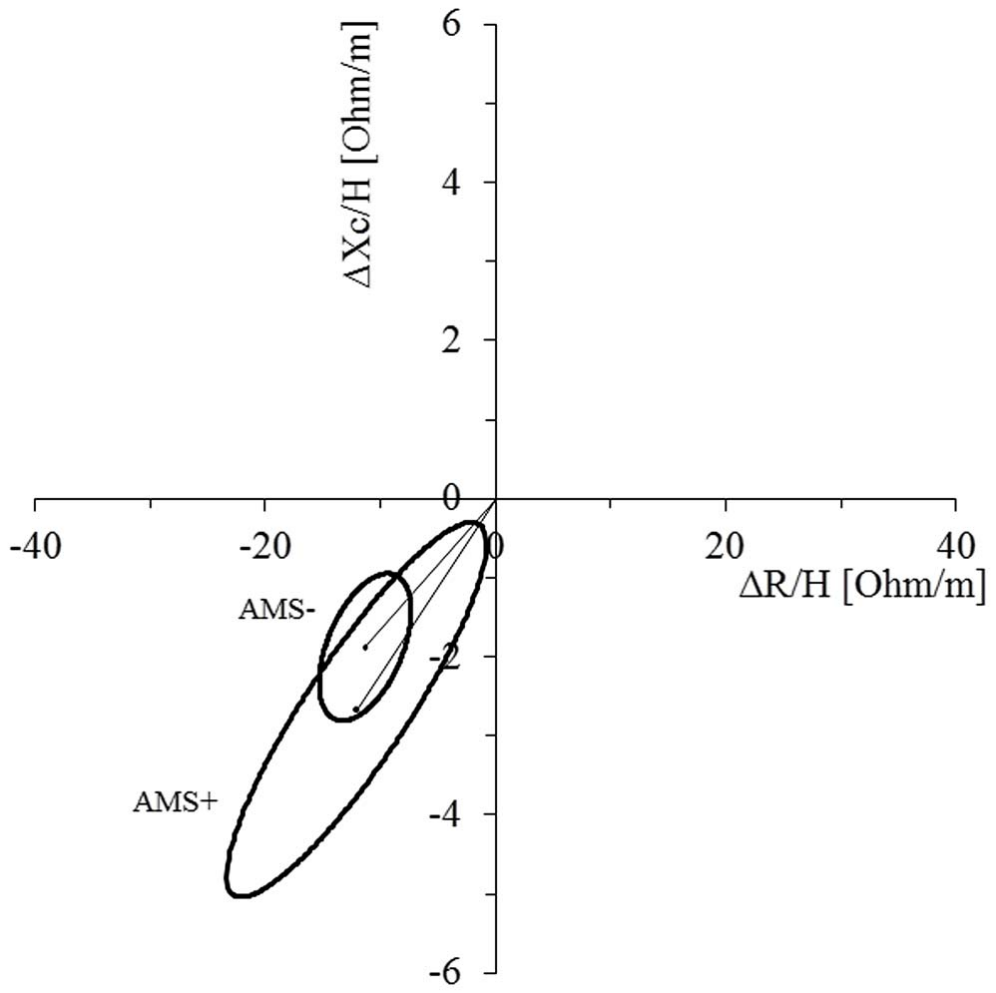
Changes in the vector length and the 95% confidence ellipses of the AMS+ and AMS− group from before to after the hypoxia exposure. In both groups a significant shortening of the vector was present (p<0.001, paired one sample Hotelling's T^2^ test) indicating fluid gain [22]. There were no differences between groups (two sample Hotelling's T^2^ test).

Vectors of both AMS+ and AMS− showed a shortening (p<0.001) (Figure 2). Overall changes (Δ) in vector length correlated significantly with changes in weight (r = −0.550, p<0.001), fluid intake (r = −0.533, p<0.001) and net water balance (r = −0.590, p<0.001). No correlation was found between any of the BIA values (i.e. Δ vector length, Δ R, Δ Xc) and Δ Posm, Δ Na^+^, Δ Hct and estimated Δ PV.

## Discussion

The main outcomes of the present investigation were that a net gain in water balance due to a low fluid loss (water retention) but not due to the amount of fluid intake discriminated participants who during the early hours of a passive normobaric hypoxia exposure developed a severe disease state (LLS ≥ 4). Furthermore, outcomes show that each 1 ml/kg/h of water gain leads to a 3.1 times higher, while every 1 ml/kg/h increase of fluid loss to a 45% reduced probability to develop AMS. Moreover, estimates of TBW and impedance vector displacements (Figure 2) show whole body fluid gain for both, AMS− and AMS+, whereas solely the AMS+ group showed fluid shifts into the intravascular system (indicated by Δ Posm and estimated Δ PV differences).

Only a limited number of studies investigated the influence of the body hydration status on the development of AMS with inconsistent findings. When considering solely fluid intake, Basnyat et al. and Mairer et al. found that low fluid intake was associated with AMS [6], [9]. In contrast to this finding but in agreement with others [14], [15], the present results do not support that low fluid intake was linked with AMS. Examination of net balance of fluid intake and fluid loss as well as the amount of fluid loss reveals their importance in the development of AMS compared to fluid intake alone. Accordingly, both, net water balance and the amount of fluid loss are strong predictors whether getting sick or not (Nagelkerkes r^2^ = 0.532). Even though Aoki et al. did not find body hydration to be a factor for the development of AMS [13], most studies found water retention to be linked to the onset of AMS symptoms [14], [18], [33], [34]. This water retention was shown to occur already within the first 3 hours of hypoxic exposure [18] indicating that reduced fluid loss occurs ahead of AMS symptoms. The mechanisms explaining the effects of fluid retention on AMS development are not established. Some proposed effects include acute hypoxia-induced elevations of circulating arginine vasopressin levels [12], [14], [18], increased aldosterone, and atrial natriuretic peptide levels [14] that might cause anti-diuresis and/or water redistribution [14], [12]. Importantly, bioimpedance and the R-Xc graph demonstrated a significant shortening of the vector that indicates fluid retention (Figure 2). This shortening was related to Δ body mass and parameters of fluid intake and output, but not with AMS symptoms or Δ Posm. As BIVA reflects whole body hydration status the finding of a significant correlation between Δ Posm and Δ Na^+^ with AMS and the larger PV expansion in AMS+ could mean that fluid shifts into the intravascular system were present. The findings of an estimated 0.3±0.3 L increase of plasma volume in the AMS+ group (Table 2) along with the finding that TBW and ECW changes only differed by this volume (i.e., 0.3 L, Table 2) between AMS+ and AMS− supports this finding. Accordingly, Westerterp et al. found that subjects developing AMS showed the largest extracellular water shifts, even though not always in the same direction [34]. Furthermore, Loeppky et al. reported increased peripheral blood flow and vasodilation in subjects with AMS [35].

The link of the peripheral responses (i.e. fluid retention and/or fluid shifts) to the development of AMS remains unexplained but increased extracellular water levels, beside other factors (e.g. changed endothelial permeability, up-regulation of inducible nitric oxide synthase), could influence BBB permeability and might increase intracranial pressure that causes symptoms of AMS [12]. Furthermore, inadequate diuresis might lessen excretion of bicarbonate, which otherwise would compensate for the respiratory alkalosis [36]. This results in a decrease in ventilation and as a consequence lower SpO_2_ which impacts AMS development [37]. Accordingly we found lower HCO_3_^−^ and higher SpO_2_ values in AMS−, an association between HCO_3_^−^, PCO_2_ and SpO_2_ and an association between SpO_2_, HCO_3_^−^ and PCO_2_ with the AMS score. A direct association between these parameters and parameters of body hydration, however, could not be detected.

Some issues have to be addressed when interpreting the outcomes of the study at hand. Present findings only hold true for normobaric hypoxia conditions, as both, reduced barometric pressure and reduced oxygen availability contribute to fluid retention [20]. Nonetheless this study adds information on the effects of hypoxia alone on fluid balance and connects these findings to the early development of AMS. Even though overall sample size was large AMS+ group involved only 11 participants, which could have influenced statistical outcomes. Not excluding subjects with emesis would have increased AMS+ sample size but as we were not able to determine volume of emesis, this procedure seemed adequate as emesis would have influenced hydration markers. As we did not perform multiple measurements prior to the development of AMS symptoms it is not possible to state whether water retention was cause or effect of AMS. Yet Loeppky et al. showed that water retention starts within the first 3 hours of hypoxic exposure in subjects that afterwards develop AMS suggesting that water retention occurs ahead of AMS symptoms [18]. Moreover, TBW and ECW were estimated from BIA values by using equations. It has to be mentioned that such formulas are depending on hydration status which obviously was changed and therefore might have influenced outcomes. Nonetheless the R-Xc graph method, shown to be a valid method for detection of body fluid volume changes independent of equations [21], [22], confirmed the water gain. Of course isotopic measurements might have been more precisely. Furthermore, we did not measure urine specific gravity as another widely used hydration parameter. Nevertheless, according to Cheuvront et al., Posm is the only useful marker for static dehydration assessment and as accurate as urine specific gravity in the setting of dynamic dehydration assessment [38]. Furthermore, increased ventilation in hypoxia might have enhanced insensible water loss. This partly might account for the differences between body mass changes and net water balance volumes (approximately 600 ml). Additionally, the obviously elevated Hct levels are due to a systematic measurement error of the device (approximately 10%) but reliability of measurements was high and the observed changes should be addressed correctly. In this context it should be mentioned that plasma volume changes calculated solely from Hct values might to some extent be inaccurate [32] as is the assumption that all participants have a blood volume of 77 ml/kg. Finally it has to be recognized that blood gases were obtained from capillary blood. Even though ear lobe was arterialized the sample might have contained some peripheral venous blood.

## Conclusion

In conclusion, the present findings confirm that a positive net water balance due to low fluid loss at least partly is involved in the early development of AMS under nomobaric hypoxia conditions. Furthermore data suggest that most of the water gain differences between AMS+ and AMS– occurred within the vascular system. These results argue against “forced” or “over”- hydration for the prevention of AMS. From a clinical point of view measures to avoid excess fluid retention are likely to reduce AMS symptoms. However, future studies that manipulate fluid intake are needed to rigorously establish water intake recommendations at altitude.
